# FabV/Triclosan Is an Antibiotic-Free and Cost-Effective Selection System for Efficient Maintenance of High and Medium -Copy Number Plasmids in *Escherichia coli*

**DOI:** 10.1371/journal.pone.0129547

**Published:** 2015-06-09

**Authors:** Syed A. Ali, Yik Wei Chew

**Affiliations:** Oncological and Radiological Sciences, Advanced Medical and Dental Institute, Universiti Sains Malaysia, Bertam, 13200 Kepala Batas, Pulau Pinang, Malaysia; Indian Institute of Science, INDIA

## Abstract

Antibiotic resistance genes and antibiotics are frequently used to maintain plasmid vectors in bacterial hosts such as *Escherichia coli*. Due to the risk of spread of antibiotic resistance, the regulatory authorities discourage the use of antibiotic resistance genes/antibiotics for the maintenance of plasmid vectors in certain biotechnology applications. Overexpression of *E*. *coli* endogenous *fab*I gene and subsequent selection on Triclosan has been proposed as a practical alternative to traditional antibiotic selection systems. Unfortunately, overexpression of *fab*I cannot be used to select medium –copy number plasmids, typically used for the expression of heterologous proteins in *E*. *coli*. Here we report that *Vibrio cholera* FabV, a functional homologue of *E*. *coli* FabI, can be used as a suitable marker for the selection and maintenance of both high and medium -copy number plasmid vectors in *E*. *coli*.

## Introduction

A majority of bacterial plasmid vectors contain antibiotic resistance genes as selection markers. Consequently, antibiotics are frequently used in the maintenance of the plasmid vectors in *Escherichia coli* (*E*. *coli*) [1]. However, use of antibiotics is undesirable in several biotechnology applications such as gene therapy (involving direct introduction of plasmid DNA into humans) and production of recombinant proteins intended for therapeutic purposes [2]. Moreover, for large-scale fermentation, plasmid selection using antibiotics can prove very costly [3] Drawbacks associated with antibiotic–based selection have therefore encouraged the development of various antibiotic marker-free selection approaches. Many such systems have been reported [4, 5, 6, 7, 8, 9, 10, 11] but are not extensively adopted by the labs for a variety of reasons that, among other, include use of specialized strains and/or reagents.

Triclosan, a polychloro phenoxy phenol, is a Food and Drug Administration (FDA)–approved non-antibiotic biocide agent found in various consumer products such as toothpastes, mouthwashes, soaps, shampoos, deodorants, and other cleaning supplies [12, 13]. Triclosan exerts its biocidal action by binding to bacterial enoyl-acyl carrier protein reductase enzyme (ENR), which is encoded by *fab*I gene in *Escherichia coli* (*E*. *coli*). Triclosan inhibits FabI through binding at the ACP-enoyl substrate site, forming a stable Triclosan/NAD^+^/FabI ternary complex. When overexpressed, FabI confers resistance towards Triclosan-mediated growth inhibition of *E*. *coli* [14]. Overexpression of *fab*I is thus proposed as an alternate plasmid selection marker in place of undesirable antibiotic markers such as β-lactamase.

Goh and Good were the first to report the use of FabI/Triclosan system as a plasmid selection marker [15], followed by the use of this system in their pestivirus DNA vaccine vectors [16]. Jang and Magnuson reported an improved system that employed mFabI (*fab*I gene containing G93V mutation), which conferred better resistance towards Triclosan [17]. Both groups have independently demonstrated that FabI (and mFabI)/Triclosan is a feasible plasmid selection marker system with notable advantages over traditional antibiotic-based selection markers. Although it is suggested that FabI/Triclosan would be a viable selection marker for a variety of bacterial vectors, it has not been shown experimentally.

The objective of this work was to engineer *fab*I-based medium-copy number expression vectors that could be maintained in the presence of Triclosan. However, all attempts to clone *E*. *coli* wild type *fab*I gene in the expression vector pSA-HNef-6His [18], a medium-copy number pBR322-based vector, were failed. It appeared that *fab*I, when expressed under its own promoter or under P3 promoter (of β-lactamase) on a medium-copy number (15–20) plasmid, was not producing adequate FabI enoyl-ACP reductase that would confer resistance towards Triclosan. We then turned our attention to FabI homologues that exist in other bacteria. FabL and FabK are FabI functional homologues that are present in *Bacillus subtilis* and *Streptococcus pneumonia* respectively [19, 20]. FabV is the fourth enoyl-ACP reductase isoform, identified in *vibrio* species [21]. Although a member of SDR (short chain dehydrogenase reductase) family and a functional homologue of FabI, FabL, and FabK, the FabV has little sequence homology (15–30%) among members [21]. Because we had *Vibrio cholera* available in our bacteria collection, we decided to clone *fab*V gene in the medium-copy number plasmids and evaluate as an alternative selection marker.

We exchanged β-lactamase (*bla*) gene with *fab*V in both the high (pUC19-based), and medium (pBR322 based)-copy number plasmids and compared the bacterial growth, transformation efficiency, and plasmid yield with the control plasmids expressing β-lactamase or *fab*I. Our data show that FabV/Triclosan is a suitable non-antibiotic selection system for the maintenance of both high and medium-copy number plasmids in *E*. *coli*.

## Material and Methods

### Bacterial strains and culture conditions

The *E*. *coli* strains DH5α (NEB #C2987) and JM109 (NEB #E4107) were used for cloning whereas BL21(DE3) (NEB # C2527) along with the other two *E*. *coli* strains was used for subsequent experiments. All three bacteria strains were grown aerobically in LB (Miller) broth, or on LB (Miller) agar at different temperatures, and in the presence or absence of Ampicillin (100 μg/ml) and/or Triclosan (1–10μM). Bacterial strains were stored in glycerol (50%)-supplemented LB broth at -80°C.

### Plasmid construction

PCR amplifications of DNA fragments intended for cloning purposes was carried out using Q5High-Fidelity DNA Polymerase (NEB #M0491), whereas Taq DNA Polymerase (NEB #M0273) was used for routine colony PCR. PCR amplified or restricted DNA fragments were reaction cleaned-up or gel-purified using NucleoSpin Gel and PCR Clean-up kit (Macherey-Nagel GmbH & Co #740609). Plasmid DNA was purified using Wizard Plus SV Minipreps DNA Purification System (Promega #A1465). Vector and insert were mixed in 1:3 molar ratios (unless otherwise specified) and ligated in presence of T4 DNA ligase (NEB #M0202) at 4°C for 18 h. For colony PCR, at least 10 randomly selected bacterial colonies were PCR amplified using a vector-specific and an insert-specific primer (**Table 1**). Transformants that contained an amplicon of expected size by PCR were then verified using DNA restriction and sequence analyses. Construction of various engineered vectors (see **Table 2**) is described below.

**Table 1:** Oligonucleotides used to construct vectors used in this study.

Primer name	Sequence (5’ to 3’)
Out-bla-ClaI-R	GGACATCGATACTCTTCCTTTTTCAATATTATTG
Out-bla-SmaI-F	GTAACCCGGGCTGTCAGACCAAGTTTACTCA
FabV-ClaI-F	GAGTATCGATGATCATCAAACCTAAAATTCGT
FabV-SmaI-R	ACAGCCCGGGTTACTCGATATCAATCACATCGAA
FabI-ClaI-F	GAGTATCGATGGGTTTTCTTTCCGG
FabI-SmaI-R	CAGCCCGGGTTATTTCAGTTCGAGTTCG
pSA-F	CAAGAACTGC*GAGCTC*CAC
pMXB10-up101-F	GATCCCGCGAAATTAATACG

**Table 2:** Plasmids used in this study.

No.	Vector	Essential features
1	pUC19	pMBI ori (high-copy number)
		Constitutive expression/production of *bla* under P3 promoter
2	pUC19-FabV	Derivative of pUC19 (high-copy number)
		Constitutive expression/production of *fab*V under P3 promoter
3	pUC19-FabI	Derivative of pUC19 (high-copy number)
		Constitutive expression/production of *fab*I under P3 promoter
4	pSA-HP24-6His	pBR322 ori (medium-copy number)
		Constitutive expression/production of *bla* under P3 promoter
		HIV-1 p24 (CA) expression/production under IPTG-inducible T7 promoter
5	pSA-HP24-FabV	Derivative of pSA-HP24-6His (medium-copy number)
		Constitutive expression/production of *fab*V under P3 promoter
		HIV-1 p24 (CA) expression/production under IPTG-inducible T7 promoter
6	pBR322-FabI	pBR322 ori (medium-copy number)
		Constitutive expression/production of *fa*bI under P1 and P3 promoters

#### Construction of pUC19-FabI and pBR322-FabI vectors

The 789bp *fab*I gene was PCR amplified from the genomic DNA of *E*. *coli* BL21(DE3) using FabI-*Cla*I-F/FabI-*Sma*I-R primers (**Table 1**). Restriction enzyme sites for *Cla*I and *Sma*I were introduced into pUC19 and pBR322 vectors by whole-plasmid PCR amplification using Out-bla-*Cla*I-R/Out-bla-*Sma*I-F primers (**Table 1**). The PCR amplified vectors (pUC19/pBR322) and insert (*fab*I) were restricted with *Cla*I/*Sma*I, ligated, and transformed into chemically competent DH5α cells. Transformants were selected on 1μM Triclosan–containing LB agar plates after 18 h incubation at 30°C.

#### Construction of pUC19-FabV and pSA-HP24-FabV vectors

The 1206bp *fab*V gene was PCR amplified from the genomic DNA of *V*. *cholerae* O1 biotype El Tor using FabV-*Cla*I-F/FabV-*Sma*I-R primers (**Table 1**). Whole plasmid PCR using Out-bla-*Cla*I-R/Out-bla-*Sma*I-F primers (**Table 1**) was carried out to introduce *Cla*I and *Sma*I restriction sites in pUC19 and pSA-HP24 vectors. The PCR amplified vectors (pUC19/pSA-HP24) and insert (*fab*V) were restricted with *Cla*I/*Sma*I, ligated, and transformed into chemically competent DH5α cells. Transformants were selected on 1μM Triclosan–containing LB agar plates after 18 h incubation at 30°C.

### Determination of Triclosan–mediated inhibition of bacteria

To determine Triclosan–mediated inhibition, three *E*. *coli* strains i.e. DH5α, JM109, and BL21(DE3) were cultured overnight in LB broth, diluted to 0.05 OD600 in the same medium, and grown in the absence and presence (10–0.325μM) of Triclosan at various temperatures *i*.*e*. 37°C **(A)**, 30°C **(B)**, and 22°C **(C)**, while shaking at 250rpm for 12 h. Bacterial growth was then measured by absorbing the diluted samples at 600nm and graphs plotted. Error bars show standard deviations calculated from at-least six (6) independent experiments performed in triplicate.

### Bacterial transformation/growth/plasmid DNA extraction

Bacteria transformed with FabV/FabI and Bla–plasmids were maintained on agar plates or cultured in broth containing either 1μM Triclosan or 100μg mL^-1^ Ampicillin respectively. Single colonies were obtained by streaking on LB agar plates and incubating at 37 or 30°C. Seed cultures were prepared by inoculating single colonies in 1-5mL LB broth and incubating at 37, 30, or 22°C overnight (12–16 hours) while shaking at 250rpm. For subsequent experiments, seed cultures were diluted to 0.05 OD600 in fresh media and then incubated at required temperature and length while shaking at 250rpm.

#### Effect of FabV/Triclosan selection on bacterial growth

*E*. *coli* DH5α, JM109, and BL21(DE3) were transformed with FabV (pUC19-FabV and pSA-HP24-FabV), FabI (pUC19-FabI, pBR322-FabI), or Bla (pUC19–Bla, pSA-HP24-Bla)-plasmids, and the transformants were selected on LB agar plates. Seed cultures were used to inoculate 25mL LB broth in 250mL baffled flasks and cultures grown at 37, 30, and 22°C for up to 12 hours while shaking at 250rpm. Samples were collected at one hour interval and growth was measured by absorbing the diluted samples at 600nm and graphs plotted. Error bars show standard deviations calculated from at-least six (6) independent experiments performed in triplicate.

#### Effect of FabV/Triclosan selection on plasmid DNA yield

*E*. *coli* DH5α and JM109 were transformed with high-copy number pUC19-Bla, pUC19-FabV, pUC19-FabI, and medium-copy number pSA-Hp24-Bla, pSA-Hp24-FabV, pBR322-FabI plasmids and selected on LB agar plates containing 1μM Triclosan (for FabV/FabI plasmids) and or 100μg mL^-1^ ampicillin (for Bla plasmids). Seed cultures were used to inoculated 5mL LB broth in 50mL flasks and cultured for another 18 hours at 37 or 30°C while shaking at 250rpm. Cell density was then measured by absorbing the diluted samples at 600nm and normalized to 2 OD600. One mL of the normalized culture was then used to extract the plasmid following supplied protocol. The quantity of the extracted plasmid DNA was measured by fluorometry using Qubit Fluorometer and Qubit dsDNA BR Assay Kit (Invitrogen, life technology).

#### Effect of FabV/Triclosan selection on Transformation Efficiency

Plasmid DNA extracted in the previous experiment were diluted to 100pg μL^-1^ and used to transform home-made chemically competent DH5α and JM109 cells [22]. In order to achieve high level of consistency and reproducibility, we established a medium-throughput thermocycler–based transformation assay. The chemically competent cells were dispensed at 50μL per tube in a thin-walled 0.2mL PCR 8-tube FLEX FREE strip (USA Scientific #1402–1800) and stored at -80°C until used. Thermocycler (Agilent SureCycler 8800) was programmed with following thermal settings: 45 minutes at 4°C, then 90 seconds at 42°C (heat shock step) and 5 minutes at 4°C. PCR 8-tube strips containing competent cells were placed onto the 4°C thermocycler block and the cells thawed for 10 minutes. One μL of 100pg μL-^1^ plasmid DNA was added to the competent cells. The heated lid was closed and program allowed completing, after which 100μL room temperature SOC was added to each tube and bacterial cells transferred to a 2.0mL centrifuge tube containing 850μL SOC. The cells were incubated at 30°C for 90 minutes while shaking at 250rpm and plated on LB agar plates containing appropriate selection agents. The plates were incubated at 30°C for 18 hours and number of colonies counted. Transformation efficiency (TE) was calculated using the formula: TE = Colonies/μg DNA/Dilution factor.

### Statistical analysis

The statistical analysis was done using SPSS software. Statistical analysis was carried out using the independent t-test to determine the significant differences between groups mean. Within group was used one-way ANOVA to compare between the group. The significant value was less than 0.05.

## Results

### Recombinant DNA techniques and construction of plasmids

**Fig 1:**
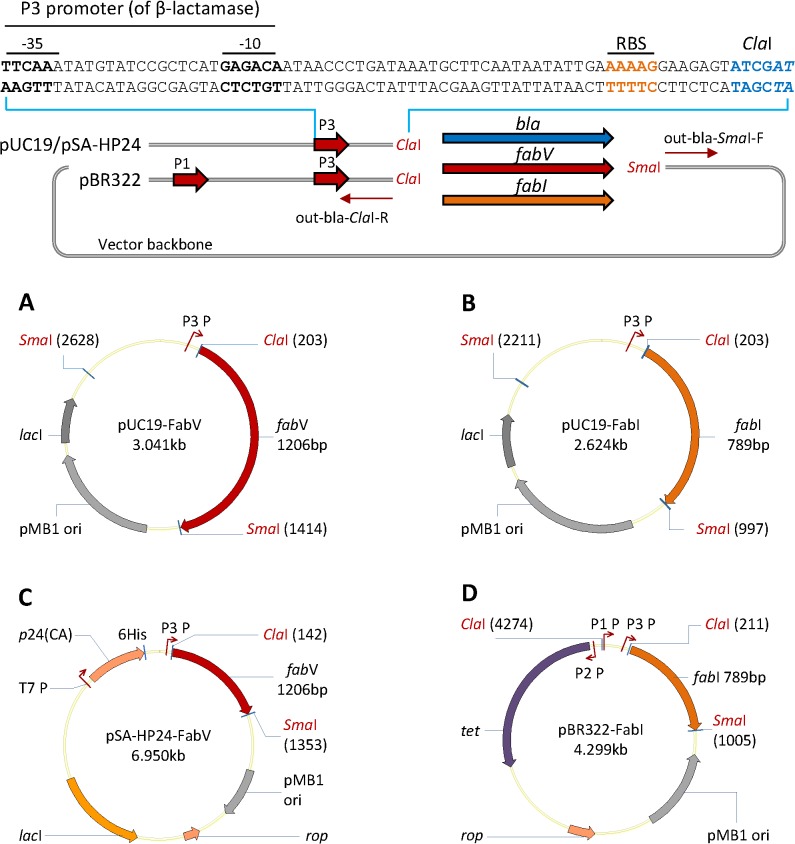
Construction of FabV and FabI-containing plasmid vectors. The *bla* gene (AmpR) was removed and restriction enzyme (*Cla*I and *Sma*I) sites were introduced downstream P3 promoter of β-lactamase by whole-plasmid PCR of pUC19, pSA-HP24, and pBR322 vectors. Inserts were prepared by PCR amplifying *fab*V and *fab*I using *Vibrio cholerae* O1 El Tor or *E*. *coli* BL21(DE3) genomic DNA respectively as DNA template. Vectors and inserts were restricted using *Cla*I and *Sma*I, gel purified, and ligated at 1:3 vector/insert ratio. These manipulations resulted in the construction of (A) FabV-containing pUC19, (B) FabI-containing pUC19, (C) FabV-containing pSA-HP24, and (D) FabI-containing pBR322 plasmid vectors.

In order to exchange *bla* (Amp^R^) gene with *fab*V or *fab*I in high-copy-number cloning (pUC19 –based) and medium-copy-number (pBR322 –based) expression (pSA-HP24-6His [18]) vectors, we PCR amplified the whole vector such that the *bla* ORF (open reading frame) was removed and restriction enzyme sites for *Cla*I and *Sma*I were generated as shown in **Fig 1 (upper panel)**. Restriction enzyme site *Cla*I also contained the first two nucleotides (AT) of the start codon. This was done to maintain the optimal distance between the start codon and the ribosomal binding site (RBS). When cloned in between *Cla*I and *Sma*I, the start codon of the inserted gene (*fab*V or *fab*I) was located at 11 nucleotides downstream of RBS (ribosomal binding site) of *bla* gene. These manipulations resulted in the construction of: **a)** pUC19-FabV; **b)** pUC19-FabI; **c)** pSA-HP24-FabV; **d)** pBR322-FabI vectors (see **Fig 1 lower panel**). In pUC19 and pSA-HP24 –derived vectors, the *bla*, *fab*V and *fab*I genes expressed under the control of a single P3 promoter of the *bla* gene, whereas in pBR322, the *fab*I gene expressed under the control of both P3 and P1 promoters as shown in **Fig 1 (upper panel)**.

### Triclosan-mediated inhibition of Bacterial Growth

**Fig 2:**
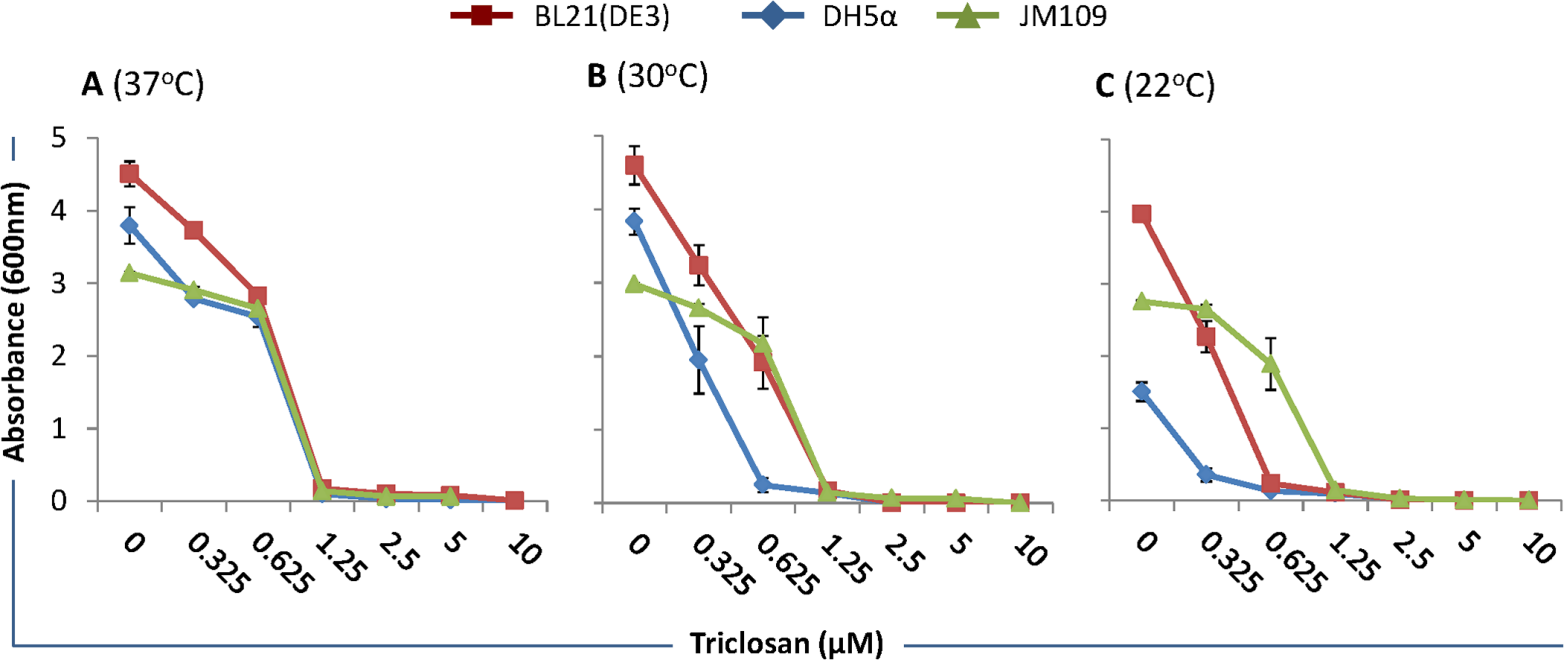
Effect of Triclosan on bacterial growth. Bacteria were cultured overnight in LB broth, diluted to 0.05 OD600 in the same medium, and grown in the absence and presence (10–0.325μM) of Triclosan at various temperatures (A) 37°C, (B) 30°C, and (C) 22°C, while shaking at 250rpm for 12h. Bacterial growth was measured by absorbing the diluted samples at 600nm and graphs plotted. Error bars show standard deviations calculated from six (6) independent experiments done in triplicate. Triclosan, at the concentration of 1.25–2.5μM, inhibited the growth of all three bacterial strains at various temperatures tested.

*E*. *coli* DH5α and JM109 are commonly used strains for DNA cloning and plasmid preparation, whereas BL21(DE3) is one of the most widely used *E*. *coli* strains for heterologous protein expression and production. We have assayed the effect of various Triclosan concentrations (0, 0.325, 0.625, 1.25, 2.5, 5, and 10uM) on these *E*. *coli* strains at 37°C, 30°C, and 22°C. Triclosan activity was tested at these temperatures because *E*. *coli* strains are grown at different temperatures for plasmid preparation and protein expression. At 37°C, the growth of all three bacterial strains was inhibited at 1.25uM Triclosan (**Fig 2A**). At 30°C, the growth of DH5α was markedly inhibited at 0.625uM Triclosan compared to JM109 and BL21(DE3), which continued to grow albeit at lower optical densities (**Fig 2B**). At 22°C, growth of both DH5α, and BL21(DE3) was inhibited at 0.625uM Triclosan but the growth of JM109 was relatively unaffected (**Fig 2C**). These data show that certain *E*. *coli* strains are more susceptible to Triclosan at lower temperatures. Triclosan, at the concentrations of 1.25–2.5μM, inhibited the growth of all three bacterial strains at 37, 30, and 22°C. Further experiments revealed that growth inhibition of all three bacterial strains at three tested temperatures was not different for 1.0uM and 1.25uM Triclosan (data not shown), and therefore 1.0uM Triclosan was used in all subsequent experiments.

### Effect of FabV/Triclosan selection system on bacterial growth

Next, we investigated the effect of FabV/Triclosan selection on the growth of DH5α, JM109, and BL21(DE3) transformed with either high (pUC–based) or medium (pBR322 –based) copy number plasmids expressing FabV as the selection marker. We transformed DH5α, JM109, and BL21(DE3) with FabV-plasmids (pUC19-FabV and pSA-HP24-FabV) and compared the growth profile with the same bacteria transformed with Bla-plasmids (pUC19-Bla, pSA-HP24-Bla), or FabI-plasmids (pUC19-FabI, pBR322-FabI) in LB broth containing Triclosan (1.0uM) or Ampicillin (100ug mL^-1^).

**Fig 3:**
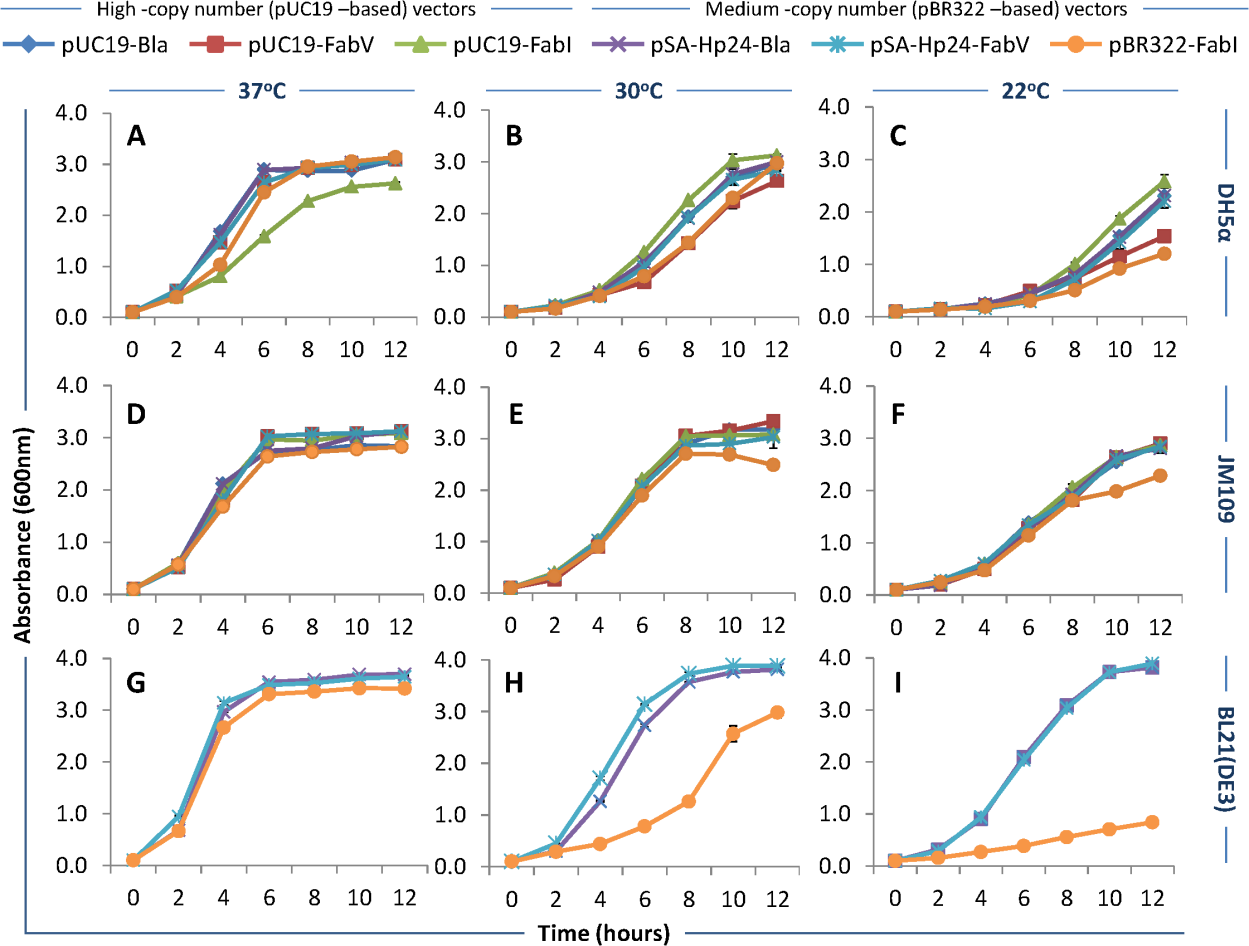
Bacterial growth characteristics. *E*. *coli* DH5α, JM109, and BL21(DE3) were transformed with FabV (pUC19-FabV and pSA-HP24-FabV), FabI (pUC19-FabI, pBR322-FabI), or Bla (pUC19–Bla, pSA-HP24-Bla)-plasmids, and the transformants were selected on LB agar plates. Seed cultures were used to inoculate 25mL LB broth in 250mL baffled flasks and cultures grown at 37, 30, and 22°C for up to 12 hours while shaking at 250rpm. Samples were collected at one hour interval and growth was measured by absorbing the diluted samples at 600nm and graphs plotted. Error bars show standard deviations calculated from at-least six (6) independent experiments performed in triplicate.

The growth of DH5α transformed with various plasmids was similar at 37°C, except for DH5α /pUC19-FabI, which showed a noticeable lag in growth, and reached to stationary phase at ~10 hours compared to 6 hours for other cultures **(Fig 3A)**. At 30°C, the growth of DH5α transformed with various plasmids was comparable, though bacteria transformed with pUC19-FabV and pBR322-FabI appeared to grow slower and yielded lower final ODs **(Fig 3B)**. At 22°C, DH5α transformed with pUC19-FabV and pBR322-FabI yielded lowest ODs compared to DH5α transformed with other plasmids **(Fig 3C)**.

The growth of JM109 transformed with various plasmids was similar and comparable with the growth of DH5a at 37°C. However, unlike DH5α, JM109/pUC19-FabI growth was indifferent from other cultures **(Fig 3D)**. At 30°C, the growth of various cultures was similar and comparable with DH5α for the same temperature **(Fig 3E)**. At 22°C, JM109 grew noticeably better compared to DH5α at the same temperature, and growth patterns were similar between JM109 transformed with various plasmids **(Fig 3F)**.

At 37°C, BL21(DE3) transformed with various plasmids grew similarly, and as expected, reached to the stationary phase earlier (at 4 hours compared to 8 hours) compared to DH5α and JM109 **(Fig 3G)**. At 30°C, the growth pattern of BL21(DE3) transformed with pSA-HP24-FabV was comparable to the same bacteria transformed with pSA-HP24-Bla. However, BL21(DE3) containing pBR322-FabI grew noticeably slowly and to lower final cell densities **(Fig 3H)**. At 22°C, the BL21(DE3) transformed with pSA-HP24-FabV and pSA-HP24-Bla grew similarly, though relatively slowly compared to 30°C. BL21(DE3) transformed with pBR322-FabI grew at substantially lower rates and much lower final cell densities **(Fig 3I)** compared to the cultures grown at 37 and 30°C.

Together, these data show that: **a)** FabV/Triclosan selection did not alter the growth profile of JM109 transformed with both high (pUC19 –based) and medium-copy number (pBR322 –based) FabV-plasmids when grown at various temperatures; **b)** DH5α transformed with medium-copy number FabV or FabI plasmid grew slowly and to lower final ODs at 22°C; **c)** the growth profile of BL21(DE3) harbouring pBR322 –based FabV plasmids was comparable with those that contained Bla-plasmids at all three temperatures; **d)** BL21(DE3) transformed with medium-copy number FabI plasmids grew slowly and to much lower final ODs at 30 and 22°C compared to FabV and Bla vectors.

### Effect of FabV/Triclosan selection on plasmid DNA yield

Next, we studied the effect of FabV/Triclosan selection on the plasmid DNA yield from DH5α and JM109. BL21(DE3), being a recA^+^/endA^+^ strain is not recommended for plasmid preparation and was therefore omitted in these experiments. Both DH5α and JM109 were transformed with various plasmids as described in Materials and Methods and grown at 37 and 30°C in LB broth supplemented with 1.0uM Triclosan (for FabV and FabI plasmids) and 100ug mL^-1^ Ampicillin (for Bla plasmids). Cells were grown for 16 hours, and equalized amount of cells (by adjusting the OD600) was used to extract plasmid DNA.

**Fig 4:**
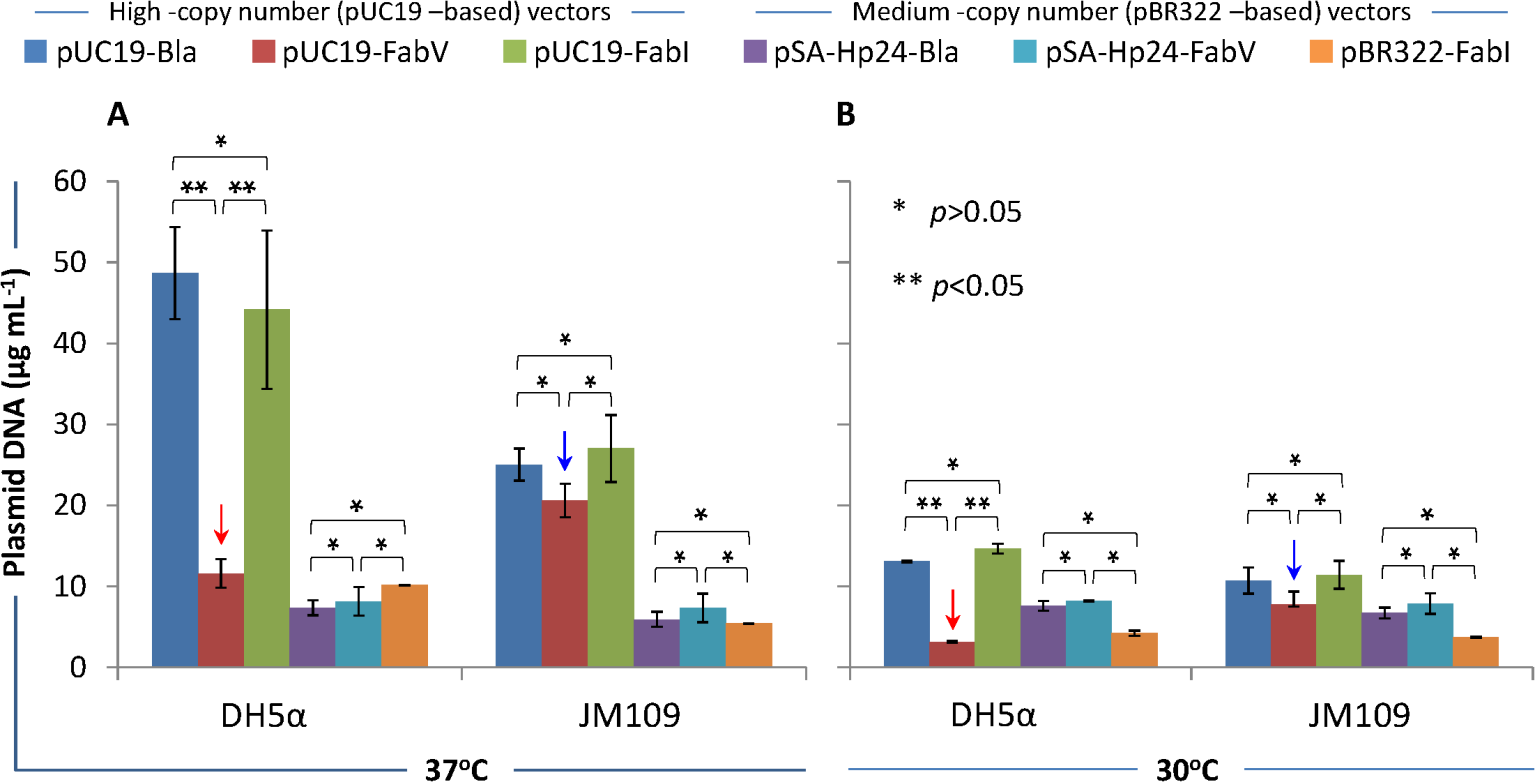
Plasmid DNA yield. *E*. *coli* DH5α and JM109 were transformed with high-copy number pUC19-Bla, pUC19-FabV, pUC19-FabI, and medium-copy number pSA-Hp24-Bla, pSA-Hp24-FabV, pBR322-FabI plasmids and selected on LB agar plates containing 1μM Triclosan (for FabV/FabI plasmids) and or 100μg mL^-1^ ampicillin (for Bla plasmids). Seed cultures were used to inoculated 5mL LB broth in 50mL flasks and cultured for another 18 hours at 37 or 30°C while shaking at 250rpm. Cell density was then measured by absorbing the diluted samples at 600nm and normalized to 2 OD600. One mL of the normalized culture was then used to extract the plasmid following supplied protocol. The quantity of the extracted plasmid DNA was measured by fluorometry using Qubit Fluorometer and Qubit dsDNA BR Assay Kit (Invitrogen, life technology). Fig 4A shows plasmid DNA yield when bacteria were grown at 37°C, whereas Fig 4B shows the plasmid DNA yield from cultures incubated at 30°C. Error bars show standard deviations calculated from at-least six (6) independent experiments performed in triplicate.

For high copy number plasmids, similar amounts of pUC19-Bla and pUC19-FabI were obtained from DH5α grown at 37 or 30°C, though almost 2.5 fold more plasmid DNA harvested from cultures cultivated at 37°C **(Fig 4A and 4B)**. Significantly (p<0.05) less pUC19-FabV recovered from DH5α cells at both 37 and 30°C (red arrows) **(Fig 4A and 4B)**. In contrast to lower plasmid DNA yield in DH5α, pUC19-FabV recovered in similar amounts (blue arrow) compared to pUC19-Bla and pUC19-FabI in JM109 at both 37 and 30°C **(Fig 4A and 4B)**. The medium-copy number pSA-HP24-Bla, pSA-HP24-FabV, and pBR322-FabI recovered in statistically similar (p>0.05) amounts from both DH5α and JM109 at 37 or 30°C **(Fig 4A and 4B)**. We titrated the amount of Triclosan (from 1.0uM to 20uM) to see if this would improve the pUC19-FabV plasmid yield in DH5α. However, increasing the amount of Triclosan had no impact on the plasmid yield (data not shown).

Together, these data show that: **a)** FabV/Triclosan selection can significantly (and negatively) affect high-copy number plasmid yield in DH5α cells; **b)** It appears that JM109 cells were refractory to this negative impact and therefore plasmid DNA yield was largely unaffected by the FabV/Triclosan selection; **c)** FabV/Triclosan is a suitable selection system for both maintenance and propagation of medium-copy number plasmids in DH5α and JM109.

### Effect of FabV/Triclosan selection on transformation efficiency

**Fig 5:**
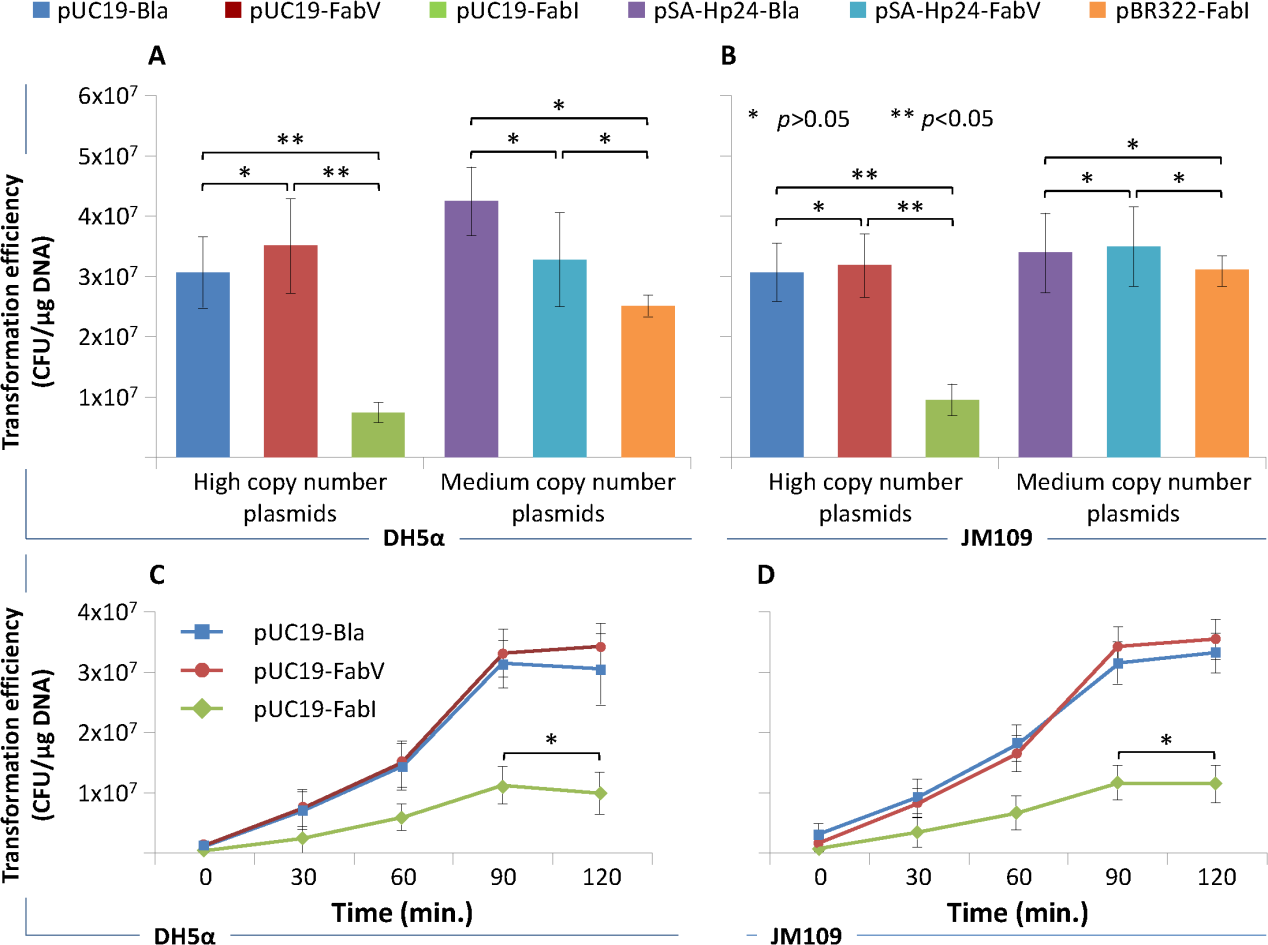
Bacterial transformation efficiency. Chemically competent *E*. *coli* DH5α and JM109 cells were transformed with 100pg μL^-1^ of purified high-copy number pUC19-Bla, pUC19-FabV, pUC19-FabI, and medium-copy number pSA-Hp24-Bla, pSA-Hp24-FabV, pBR322-FabI plasmids and selected on LB agar plates containing 1μM Triclosan (for FabV/FabI plasmids) and or 100μg mL^-1^ ampicillin (for Bla plasmids) after 18 hours of incubation at 30°C and transformation efficiency calculated. Fig 5A and B show transformation efficiency of various plasmids in DH5α and JM109 respectively. Fig 5C and D show the effect of incubation time prior to plating the transformants on selection plates. Error bars show standard deviations calculated from at-least six (6) independent experiments performed in triplicate.

We next evaluated the transformation efficiency of FabV plasmids and compared it with Bla and FabI plasmids in both DH5α and JM109. As it can be seen from **Fig 5A and 5B**, the high-copy number pUC19-FabV transformed DH5α and JM109 as efficiently as the control plasmid pUC19-Bla. However, pUC19-FabI was transformed three folds less efficiently compared to pUC19-Bla and pUC19-FabV. Transformation efficiencies of medium-copy number plasmids pSA-HP24-Bla, pSA-HP24-FabV, and pBR322-FabI were similar in both DH5α and JM109 (**Fig 5A and 5B**). We were surprised to see low transformation efficiency for bacterial transformed with pUC19-FabI. In order to confirm these results and to optimize the transformation protocol, we incubated transformed bacteria for 30, 60, 90, and 120 minutes prior to plating on the selection plates. However, decreasing or increasing the incubation time did no improve transformation efficiency of the pUC19-FabI both in DH5α and JM109 (**Fig 5C and 5D**).

**Fig 6:**
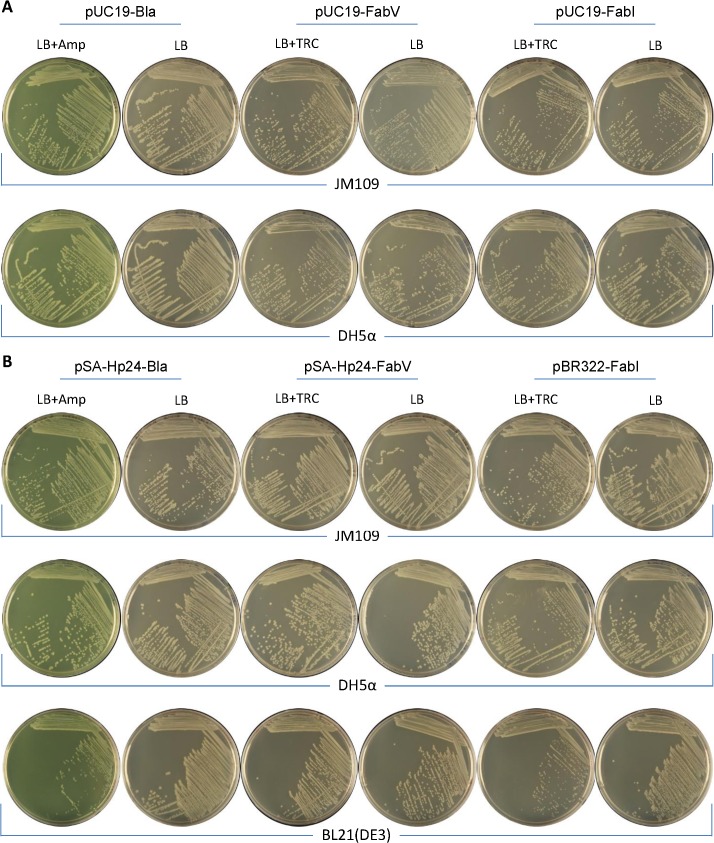
Morphology of transformed bacteria. *E*. *coli* DH5α and JM109 transformed with (A) high-copy number pUC19-Bla, pUC19-FabV, or pUC19-FabI plasmids or (B) medium–copy number pMXB-p24-Bla, pMXBp24-FabV, or pBR322-FabI plasmids were incubated at 30°C for 18 hours on plates with our without selection agent (Ampicillin or Triclosan) and photographed. No appreciable differences noted when the transformants were plated on Agar plates containing selection agent or not.

DH5α and JM109 transformed with high-copy number pUC19-Bla, pUC19-FabV, or pUC19-FabI produced similar sized (~3.5mm) colonies following 18 hours incubation at 30°C. The colonies were of uniform size and morphology as shown in **Fig 6A**. Similarly, DH5α and JM109, and BL21(DE3) transformed with medium–copy number pMXB-p24-Bla, pMXBp24-FabV, or pBR322-FabI produced colonies of similar size and morphology (**Fig 6B**). Taken together, these results show that FabV/Triclosan is comparable with traditional *bla*/Ampicillin plasmid selection system in terms of transformation efficiency and does not exhibit noticeable adverse effects on the growth of the transformed bacteria.

## Discussion

Here we are reporting a non-antibiotics selection system that is based on the overexpression of *fab*V gene of *V*. *cholera* and subsequent selection of *fab*V overexpressing bacteria in culture media supplemented with Triclosan. Two groups have independently demonstrated the efficacy of *fab*I/Triclosan selection system [15, 16, 17]; however, whether *fab*I/Triclosan selection system could also be used for the maintenance of medium-copy number plasmid vectors typically used for heterologous proteins expression in *E*. *coli* was not shown.

We attempted to exchange *bla* gene in pBR322 –based expression plasmids with the wild type *fab*I gene (only open reading frame and no promoter) of *E*. *coli*. However, we were unable to select clones on agar plates containing Triclosan. Next, we tried to clone *fab*I gene together with its own promoter but still could not get any colony on the selection plates. In contrast, we experienced no problem cloning *fab*I gene in the high-copy number pUC–based vectors. These data lead us to hypothesize that *fab*I, when expressed under its own promoter or under a weak P3 promoter (of *bla* gene) from a medium-copy number pBR322 –based expression plasmids, did not produce sufficient enoyl-ACP reductase that would confer resistance towards 1μM Triclosan present in the medium. To test our hypothesis, we exchanged the *bla* gene in pBR322 vector with *fab*I. Our hypothesis proved correct when we successfully managed to clone the *fab*I gene in pBR322 vector, in which the *fab*I was expressed under the control of two promoters i.e., P3 and P1 promoters. P3 is the natural promoter of *bla* gene, whereas P1 was artificially generated during the process of ligation of two different DNA fragments to create pBR322 [23].

However, we were not interested in expressing *fab*I under dual weak or a single strong promoter because this approach would result in the excessive consumption of available precursor metabolites, and energy for the synthesis of FabI. Consequently, the more selection marker produced the less that these resources are available for the synthesis of target heterologous protein in the host bacteria. Aside from creating metabolic burden, excess marker synthesis will result in substantial contamination of the expressed heterologous protein with the selectable marker protein and selective agent.

FabV, encoded by *fab*V gene of *V*. *cholera*, is unlike the other SDR ENRs in that it is refractory to Triclosan inhibition, is 60% larger than the typical SDR family member (which are generally about 250 residues long), and has an eight-residue space between the active-site tyrosine and lysine residues (Tyr-X8-Lys) [21]. It is also reported that the expression of FabV from a single copy plasmid in *E*. *coli* made the host resistant to Triclosan [21]. Indeed, when expressed under P3 promoter from pBR322 –based plasmids, we were able to select colonies on Triclosan–supplemented Agar plates.

Since the overexpression of FabV as a selection marker has not been evaluated previously, we carried out an exhaustive characterization of FabV/Triclosan selection system. Commonly used *E*. *coli* strains for molecular cloning and protein expression were employed. All three strains were susceptible to Triclosan–mediated growth inhibition at 1uM Triclosan. However, BL21(DE3) appeared to be more resistant towards Triclosan at 37 and 30°C compared to DH5α and JM109, whereas at 22°C, both BL21 and JM109 appeared to be more resistant compared to DH5α. This may be due to altered formation of stable Triclosan/NAD^+^/FabI ternary complex at different incubation temperatures, though it remains to be proven experimentally.

When these bacterial strains were transformed with Bla, FabI, and FabV plasmids, JM109 appeared to be least affected by the overexpression of FabV and in the presence of 1μM Triclosan at all three tested temperatures. Whereas no significant differences in growth observed at 37°C for all three bacterial strains, the FabI–expressing BL21(DE3) clearly exhibited diminished growth at 30°C and 22°C. Further studies are needed to explain why over-expression of FabV, a larger and non-endogenous (with respect to *E*. *coli*) protein did not affect the growth of BL21(DE3) at 30 and 22°C.

DH5α and JM109 are two commonly used *E*. *coli* strains for the propagation and purification of plasmid DNA. DH5α yielded significantly higher amounts of high-copy number plasmids at both 37 and 30°C compared to JM109. However, high-copy number plasmid containing FabV as selection marker yielded in low amounts compared to plasmids with Bla and FabI selection markers when isolated from DH5α. This was in contrast to JM109 cells that yielded similar amounts of plasmid DNA irrespective of selection marker. These variances in plasmid DNA yield were not due to the bacterial biomass that appeared to be similar following 16 hours of incubation. Moreover, the biomass was normalized before the isolation of the plasmid DNA. In contrast to high-copy number plasmids, we did not observe statistically significant differences in yields between the medium-copy number plasmids containing Bla, FabV, or FabI selection markers at both 37 and 30°C. These data suggest that JM109 but not DH5α is a suitable *E*. *coli* host for the propagation and purification of high-copy number plasmids harbouring FabV as selection marker. We have also assessed the quality of the plasmid DNA by restriction digestion (data not shown) and found that all plasmid DNA were restricted efficiently irrespective of selection makers used, bacterial host, and culture temperature.

We have assessed the transformation efficiency of various plasmids in both DH5α and JM109 cells. Compared to high-copy number Bla and FabV plasmids, FabI plasmids transfected least efficiently in both DH5α and JM109 cells. It is difficult to explain these results, for FabI plasmids yielded in amounts comparable to Bla plasmids from DH5α and JM109 cells in the previous experiment. It was possible that 90 minutes incubation at 30°C was not sufficient for bacteria to produce sufficient FabI that would confer resistance to Triclosan following spreading on the selection plates. In order to rule out this possibility, we incubated the transformed bacteria (after heat shock) for 30, 60, 90, and 120 minutes before plating the transformants on the selection plates. However, there was no statistically significant difference in the number of transformant obtained after 90 or 120 minute incubation. In contrast, no significant differences were noted in transfection efficiencies for medium–copy number plasmids harbouring various selection markers.

Previous report suggested that overexpression of FabI in the absence of Triclosan is toxic towards the bacteria. We streaked the bacteria transformed with both high and medium–copy number plasmids harbouring Bla, FabV, and FabI on LB agar plates with our without selection agents and compared the colony size and morphology after 18 hours incubation. In contrast to earlier reports, we did not observe an appreciable difference in the size or morphology of the colonies on agar plates with or without selection marker. A possible explanation of this difference could be the strength of the promoter that was used to express the *fab*I gene. For instance in Goh & Good paper [15], pFab plasmid is described that is derived from pUC19 and contains *fab*I in place of the *bla* gene. The *fab*I gene is cloned together with its own promoter between *Ssp*I (upstream of *bla* and native to pUC19) and *Eag*I (introduced downstream *bla* gene). The P3 promoter of the *bla* gene is located upstream *Ssp*I and therefore retained in the pFab plasmid. We understand that in pFab plasmid, FabI is expressed both under its own promoter and P3 promoter. Coupled with high-copy number of pFab, we hypothesize that FabI is expressed in amounts toxic to *E*. *coli* in the absence of Triclosan in the medium.

In conclusion, this study describes the use of FabV gene as a suitable selection marker for both high and medium-copy number plasmids. FabV/Triclosan selection system is particularly suitable for the maintenance of medium-copy number plasmids, especially the so-called expression plasmids used in heterologous proteins expression. Unlike several other non-antibiotic selection systems that require the use of specialized strains or reagents, FabV/Triclosan can easily be adapted by replacing the antibiotic genes with the FabV gene using the whole plasmid PCR as described. The final concentration of Triclosan used in 1L of culture is just 0.289mg (to give final concentration of 1μM), in contrast to Ampicillin that is used at 100mg/L, which makes this system significantly economical compared to *bla*/Ampicillin.
